# Ezetimibe prescriptions in older Canadian adults after an acute myocardial infarction: a population-based cohort study

**DOI:** 10.1186/s12944-017-0649-5

**Published:** 2018-01-08

**Authors:** Kristin K. Clemens, Salimah Z. Shariff, Eric McArthur, Robert A. Hegele

**Affiliations:** 1 St. Joseph’s Health Care London, PO BOX 5777, STN B, London, ON N6A 4V2 Canada; 2 Department of Medicine, Division of Endocrinology, Western University, London, ON Canada; 3 Institute for Clinical Evaluative Sciences, ON, Canada; 4 Lawson Health Research Institute, London, ON Canada; 5 Department of Epidemiology and Biostatistics, Western University, London, ON Canada; 6 Robarts Research Institute, 100 Perth Drive, London, ON N6K 5K8 Canada

**Keywords:** Ezetimibe, Older adults, Prescriptions, Acute myocardial infarctions

## Abstract

**Background:**

The utility of ezetimibe in preventing cardiovascular outcomes remains controversial. To guide future assessments of the effectiveness of ezetimibe in routine care, we evaluated how this medication has been prescribed to high-risk older adults in Ontario, Canada.

**Methods:**

Using linked healthcare databases, we carried out a population-based cohort study of older adults who were discharged from hospital following an acute myocardial infarction from 2005 until 2014. We ascertained the rate of ezetimibe initiation within 6 months of their discharge. We also examined the characteristics of new ezetimibe prescriptions, as well as the predictors for receiving the therapy.

**Results:**

Seventy one thousand one hundred twenty five older adults were hospitalized for an acute myocardial infarction between 2005 and 2014 (mean age 78.36 ± 7.71 years, 45.8% women). Only 1230 (1.7%) patients were newly prescribed ezetimibe within 6 months of their hospital discharge. The median duration of continuous use of ezetimibe was 1.2 years (IQR 0.3–3.5 years). Ezetimibe was prescribed more often to patients living in rural areas, with a history of coronary artery disease, on high-potency statins, and, with evidence of healthcare follow-up after hospital discharge. Prescriptions were less common in men, older patients, those living in long-term care facilities, those with a history of congestive heart failure, and those who were hospitalized for a myocardial infarction in more recent years.

**Conclusions:**

Real-world drug effectiveness studies can help to complement the findings of randomized controlled trials. In our region however, only a small proportion of high-risk older adults received a prescription for ezetimibe following a myocardial infarction. Clinical and research implications are discussed.

**Electronic supplementary material:**

The online version of this article (10.1186/s12944-017-0649-5) contains supplementary material, which is available to authorized users.

## Background

Given their proven efficacy in lowering low-density lipoprotein cholesterol (LDL-C) and reducing cardiovascular events and mortality, statins are first-line therapy for the treatment of dyslipidemia [1]. Despite their efficacy however, some patients experience side effects from statins [2], and are unable to attain their lipid targets [3–6]. Although recent clinical trial evidence supports the selective use of non-statin agents for cardiovascular risk reduction [7], this has not always been the case in practice, particularly for ezetimibe.

Ezetimibe reduces cholesterol absorption in the small intestine by targeting the Niemann-Pick C1 like 1 protein [8]. It is well-tolerated, and reduces LDL-C by about 20% as monotherapy, and by an additional 18–24% when used alongside statin therapy [9, 10]. Because of its LDL-C lowering potential, in 2002 the United States Food and Drug Administration (FDA) and Health Canada, approved ezetimibe as a second-line medication for people with statin intolerance or in those unable to reach their lipid targets on statins alone. In 2003, ezetimibe entered the Canadian market, and in 2004, it was listed on Ontario’s drug benefits formulary.

Although ezetimibe lowers the reliable surrogate marker of LDL-C, its utility in preventing clinically important cardiovascular outcomes has remained controversial for more than a decade [11–15]. Evidence for a possible benefit of ezetimibe has been observed more recently in the Study of Heart and Renal Protection (SHARP) trial [16], and in the IMProved Reduction of Outcomes: Vytorin Efficacy International Trial (IMPROVE-IT) [10]. A recent pharmaco-economic modeling effort has also suggested that if statin monotherapy was insufficient to lower LDL-C, ezetimibe might be the next drug to add [17]. As ezetimibe is an inexpensive, easy to ingest, and well-tolerated medication, further evaluations of its effectiveness are necessary to guide prescribing [18].

Well-conducted, population-based drug studies can help to complement the findings of randomized controlled trials [19]. Although observational studies are considered lower on the pyramid of medical evidence, they can produce results that are more generalizable than clinical trials as they include a broader range of patients, many of whom would not meet the strict inclusion criteria of clinical trials. Observational drug studies can also help to ascertain how effective medications are in a “real-world” setting [19, 20].

Before executing such studies however, it is important to confirm study feasibility by quantifying patterns in drug prescriptions in routine care. In the current study, we aimed to examine how often ezetimibe has been prescribed to older adults following a hospitalization with an acute myocardial infarction (AMI) in Canada’s most populous province (Ontario). We also examined the predictors of new ezetimibe prescriptions.

## Methods

### Design and setting

We conducted a population-based retrospective cohort study to describe the frequency of new use of ezetimibe among older adults following a hospitalization for an AMI in Ontario, Canada. Ontario is Canada’s largest province with a population estimate of 13.9 million residents. In Ontario, people who are 65 years and older have universal healthcare including access to medications, hospital, diagnostic and physician services. Information on their use of these services is collected and maintained in the records of administrative databases held at the Institute for Clinical Evaluative Sciences (ICES). Databases are linked using unique encoded identifiers.

Our study analysis was completed at ICES according to a pre-specified protocol (available from the authors upon request). We followed the guidelines for the reporting of studies using routinely collected healthcare data (RECORD) (Additional file 1) [21].

### Sources of data

We ascertained baseline characteristics and outcomes using the records from several databases.

We used the Registered Persons Database of Ontario to collect vital statistics. This database contains demographic information on anyone who has ever received a healthcard in our province. We used the Canadian Institute for Health Information’s Discharge Abstract Database and the National Ambulatory Care Reporting System database to examine the medical comorbidities of patients. These databases contain patient diagnoses coded during inpatient and emergency room encounters, respectively. The diabetes and hypertension status of patients were collected from the Ontario Diabetes Database (ODD) and the Hypertension Database (HYPER). These databases were derived using validated coding algorithms (sensitivity of ODD 86% and specificity 97%; sensitivity of HYPER 73% and specificity 95%) [22, 23]. We used the Ontario Drug Benefit (ODB) database to capture prescription medications. This database contains records of all prescriptions dispensed to people age 65 and older with an error rate of less than 1% [24]. Limited use (LU) codes (codes required to prescribe drugs that are not listed under the general drug benefits formulary) [25], were ascertained from the ODB database. These codes were used to determine the reasons for new ezetimibe prescriptions (i.e. statin intolerance or contraindication to statin therapy [LU code 381], or failure to meet lipid targets on statin monotherapy [LU code 380]). We also used the drug identification number (DIN) database (IMS Brogan Inc., Mississauga, ON) to capture the dose of statin medications. Further, we examined the characteristics of physicians who prescribed ezetimibe through the ICES Physician Database (IPDB), and determined if patients were rostered to a family physician with the Client Agency Program Enrolment (CAPE) database. Physician visits, laboratory services, and additional medical comorbidities and procedures were obtained from the Ontario Health Insurance Plan (OHIP) database, which contains physician diagnostic and billing codes.

We used International Classification of Diseases 9^th^ Revision (ICD-9, pre-2002), 10th Revision (ICD-10, 2002+), Canadian Classification of Diagnostic, Therapeutic, and Surgical Procedures (CCP, pre-2002), Canadian Classification of Health Interventions (CCI, 2002+), and OHIP billing and diagnostic codes to assess baseline comorbidities in the 5 years prior to their hospitalization for an AMI. Health services utilization was examined in the 1 year prior to their hospitalization (coding definitions provided in Additional file 2).

### Patients

We included all patients with a valid healthcard number who had evidence of a hospitalization for an AMI (defined by ICD 10 code I21) between April 1, 2005 and March 31, 2014 in Ontario (the administrative [i.e. fiscal] years of 2005 to 2013). The following patients were then excluded: 1) those with a missing sex or age, and those < 66 years; 2) those who were not residents of Ontario at hospital admission; 3) those who died prior to their hospital discharge; and 4) those with evidence of a hospitalization for an AMI in the 5 years prior to cohort entry (to limit our cohort to patients who did not have an AMI in recent years). If patients had more than one hospitalization for an AMI over the study period, we examined their first hospitalization only. We also excluded patients with evidence of a prescription for ezetimibe in the 6 months prior to their hospitalization, to capture new ezetimibe prescriptions following their first myocardial infarction within the study period.

### Outcomes

Our primary aim was to determine the percentage of patients with a new prescription for ezetimibe within 6 months of their hospital discharge. We chose 6 months of follow-up as we judged this to be a reasonable period of time in which high-risk patients would have lipid-lowering therapies initiated, titrated or changed to meet their LDL-C targets. In additional analyses, we detailed the characteristics of the new prescriptions (e.g. reason for ezetimibe prescription as determined by LU codes). We also ascertained the time between hospital discharge and a new prescription for ezetimibe, and we examined the duration of continuous use of ezetimibe, which was defined by the total number of days that the therapy was prescribed to patients allowing for a grace period between repeat prescriptions (1.5 times the days’ supply of the previous prescription). We further examined the predictors for a new ezetimibe prescription.

### Statistical analysis

We used descriptive statistics to present the characteristics of patients newly prescribed and not prescribed ezetimibe within 6 months of their hospital discharge. Continuous variables are presented as means ± standard deviation (SD), and medians (interquartile range [IQR]). Binary variables are presented as proportions. We evaluated between-group differences using standardized differences, for which a value > 10% is considered to be meaningful [26].

We used both univariable and multivariable logistic regression (with backward selection, *P* value < 0.05) to identify the clinically relevant predictors for a new ezetimibe prescription following hospital discharge (Additional file 3). For our multivariable analyses, all predictor variables could be included in the final logistic regression model. Results are presented as odds ratios (OR) and 95% confidence intervals (CI’s).

## Results

Between April 1, 2005 and March 31, 2014, there were 167, 817 patients with a valid healthcard number hospitalized for an AMI in Ontario. A total of 71, 125 older adults were included in our study cohort (flow diagram in Additional file 4). Their mean age was 78 ± 8 years, and 45.8% were women.

Only 1230 (1.7%) patients were newly prescribed ezetimibe within 6 months of their hospital discharge. This percentage increased after 2005 (7%), peaked in 2007 (16%), then appeared to decline to a rate of 2% in 2014 (Additional file 5).

When prescribed ezetimibe, the median number of days between hospital discharge and a new prescription was 63 (IQR 13–119 days). Patients were most often prescribed ezetimibe by their family physician (*n* = 509; 41.4%), followed by a cardiologist (*n* = 351; 28.5%). The majority were prescribed the therapy because they had not achieved their target LDL-C with statins (*n* = 888 [72.2%] were administered LU code 380). The remainder were prescribed ezetimibe in the setting of statin intolerance or a contraindication to the therapy (LU code 381). For those prescribed ezetimibe, the median duration of continuous use was 444 days (IQR 100–1266 days) or 1.2 years (IQR 0.3–3.5 years).

The characteristics of people newly prescribed and not prescribed ezetimibe within 6 months of their discharge are detailed in Table 1. Patients prescribed ezetimibe appeared younger, healthier, were less likely to have diabetes, hypertension or heart failure, and appeared more likely to be on high-intensity statins, fibrates or angiotensin receptor blockers.

**Table 1:** Characteristics of older adults newly prescribed and not prescribed ezetimibe within six months of a hospital discharge for an AMI

	No Ezetimibe Prescription *N* = 69, 895		New Ezetimibe Prescription *N* = 1230		Standardized Difference
Age	N	%	N	%	
Mean (SD)	78.42	7.72	74.84	6.43	50%
Median (IQR)	78	(72–84)	74	(69–79)	
Female	31, 995	45.8%	559	45.4%	1%
Rural location	11, 497	16.4%	224	18.2%	5%
Income quintile					
1 (lowest)	15, 454	22.1%	243	19.8%	6%
2	14, 762	21.1%	253	20.6%	1%
3	14, 028	20.1%	257	20.9%	2%
4	13, 162	18.8%	256	20.8%	5%
5 (highest)	12, 489	17.9%	221	18.0%	0%
Long-term care	3587	5.1%	< 6	< 0.5%	–
Year of hospital discharge					
2005	8390	12.0%	131	10.7%	4%
2006	7678	11.0%	174	14.1%	10%
2007	7974	11.4%	187	15.2%	11%
2008	7786	11.1%	121	9.8%	4%
2009	7537	10.8%	114	9.3%	5%
2010	7585	10.9%	127	10.3%	2%
2011	7530	10.8%	148	12.0%	4%
2012	7572	10.8%	117	9.5%	4%
2013	7843	11.2%	111	9.0%	7%
Rostered to a family physician	55, 396	79.3%	1015	82.5%	8%
Comorbidities^a^					
Coronary artery disease	27, 838	39.8%	509	41.4%	3%
Stroke/Transient ischemic attack	3012	4.3%	40	3.3%	5%
Diabetes	64, 973	93.0%	1102	89.6%	12%
Peripheral vascular disease	2199	3.1%	36	2.9%	1%
Chronic kidney disease	8809	12.6%	123	10.0%	8%
Dialysis	1080	1.5%	10	0.8%	7%
Hypertension	64, 745	92.6%	1094	88.9%	13%
Liver disease	1905	2.7%	32	2.6%	1%
Congestive heart failure	14, 634	20.9%	146	11.9%	24%
Coronary revascularization	2555	3.7%	71	5.8%	10%
Charlson comorbidity index					
0 (no hospitalizations)	46, 300	66.2%	921	74.9%	19%
1	7498	10.7%	114	9.3%	5%
2	6248	8.9%	95	7.7%	4%
3 or higher	9849	14.1%	100	8.1%	19%
Healthcare Utilization^b^					
Visit to a family physician	66, 325	94.9%	1165	94.7%	1%
Visit to cardiologist	32, 321	46.2%	559	45.4%	2%
Visit to endocrinologist	5025	7.2%	106	8.6%	5%
Visit to internist	24, 020	34.4%	393	32.0%	5%
Evidence of at least 1 cholesterol test	35, 060	50.2%	797	64.8%	30%
Baseline medications^c^					
Any statin	30, 329	43.4%	556	45.2%	4%
Statin intensity^d^					
Low	2335	3.3%	29	2.4%	5%
Moderate	20, 626	29.5%	314	25.5%	9%
High	6857	9.8%	205	16.7%	20%
Other	511	0.7%	8	0.7%	0%
Fibrates	1430	2.0%	56	4.6%	15%
Thienopyridines	1576	2.3%	21	1.7%	4%
Beta-blocker	24, 614	35.2%	438	35.6%	1%
Angiotensin converting enzyme inhibitor	25, 701	36.8%	415	33.7%	6%
Angiotensin receptor blocker	14, 131	20.2%	303	24.6%	11%

Unless indicated, data presented as number and percentage

Standardized differences > 10% are considered meaningful

Cell sizes < 6 are not presented for patient privacy

^a^Comorbidities were ascertained in the 5 years prior to their hospitalization for an AMI

^b^Healthcare utilization was ascertained in the 1 year prior to their hospitalization for an AMI

^c^Baseline medications were examined in the 120 days prior to their AMI hospitalization

^d^Intensity of statin therapy was categorized based upon guideline recommendations from the American College of Cardiology

In univariable analysis, significant positive predictors for a new ezetimibe prescription included: 1) being rostered to a family doctor; 2) use of high-intensity statin therapy; 3) having at least one lipid test post-discharge; 4) having a family physician visit within 30 days of discharge; and 5) having a specialist visit within 60 days of discharge. Patients less likely to receive a new prescription for ezetimibe were 1) older; 2) lived in long-term care; 3) had medical comorbidities including diabetes, chronic kidney disease, hypertension and congestive heart failure; and 4) had hospital admissions for AMIs in the latter years of study (Table 2).

**Table 2:** Predictors of a new ezetimibe prescription

	Univariable Analysis Odds Ratio (95% CI)	Multivariable Analysis Odds Ratio (95% CI)
Age (per year)	0.94 (0.93 to 0.94)	0.96 (0.95 to 0.96)
Sex		
Male	1.01 (0.91 to 1.14)	0.77 (0.68 to 0.86)
Female	Referent	Referent
Income Quintile		
Quintile 1 (lowest)	0.86 (0.72 to 1.02)	–
Quintile 2	0.94 (0.79 to 1.12)	–
Quintile 3	Referent	–
Quintile 4	1.06 (0.89 to 1.26)	–
Quintile 5 (highest)	0.97 (0.81 to 1.16)	–
Residence		
Long-term care	0.05 (0.02 to 0.14)	0.10 (0.03 to 0.32)
Community	Referent	Referent
Location of residence		
Rural location	1.13 (0.98 to 1.31)	1.24 (1.07 to 1.44)
Urban	Referent	Referent
Rostered to a family physician		
Yes	1.24 (1.07 to 1.43)	–
No	Referent	–
Year of acute myocardial infarction hospitalization	0.97 (0.95 to 0.99)	0.96 (0.94 to 0.98)
Hospital location		
Rural hospital	0.98 (0.84 to 1.15)	–
Urban	Referent	–
Coronary artery disease	1.07 (0.95 to 1.20)	1.25 (1.10 to 1.42)
Stroke	0.75 (0.54 to 1.03)	–
Diabetes	0.65 (0.54 to 0.79)	–
Peripheral vascular disease	0.93 (0.66 to 1.30)	–
Chronic kidney disease	0.77 (0.64 to 0.93)	–
Chronic dialysis	0.52 (0.28 to 0.98)	–
Hypertension	0.64 (0.53 to 0.77)	–
Liver disease	0.95 (0.67 to 1.36)	–
Congestive heart failure	0.51(0.43 to 0.61)	0.70 (0.58 to 0.85)
Charlson comorbidity index	0.87 (0.83 to 0.91)	0.95 (0.91 to 1.00)
Baseline statin use		
Yes	1.08 (0.96 to 1.21)	–
No	Referent	–
Baseline statin intensity		
No statin	Referent	Referent
High-intensity	1.76 (1.51 to 2.06)	1.72 (1.45 to 2.04)
Low-intensity	0.73 (0.50 to 1.06)	0.75 (0.51 to 1.09)
Moderate-intensity	0.89 (0.78 to 1.02)	0.88 (0.77 to 1.02)
Other intensity	0.92 (0.46 to 1.86)	0.94 (0.46 to 1.90)
Post-discharge lipid test	3.70 (3.25 to 4.20)	3.01 (2.63 to 3.43)
Post-discharge family physician visit within 30 days	1.31(1.11 to 1.56)	–
Post-discharge specialist visit within 60 days^a^	2.35 (1.85 to 2.98)	1.63 (1.29 to 2.08)

^a^Specialist visits included visits to endocrinologists, internists, cardiologists

In multivariable analysis, patients were more likely to receive ezetimibe if they: 1) lived in rural locations; 2) used high-intensity statin therapy; 3) had a pre-existing history of coronary artery disease; and 4) had a lipid test, or specialist visit after their discharge (Table 2). In contrast, 1) older patients; 2) long-term care residents; 3) men; 4) those with congestive heart failure; and 5) those with AMI hospitalizations in the latter years of our study were less likely to receive a new prescription for ezetimibe.

## Discussion

Although some patients do not achieve guideline recommended LDL-C targets on statin monotherapy, and intolerance to statins has been described in 27% of statin treated patients and 36% of high-risk patients [27, 28], in our province, only a small proportion of high-risk older adults were newly prescribed ezetimibe after an AMI from 2005 to 2014.

The reasons for infrequent ezetimibe prescriptions in high-risk older adults could be manifold. For one, although older adults benefit from LDL-C lowering [29, 30], there remains controversy over the utility of therapy in people over the age of 80, and in those with medical comorbidities or functional impairment [31]. Ezetimibe may have also been infrequently prescribed as older patients can experience adverse side effects with combination therapies [32]. This may have dissuaded practitioners from prescribing ezetimibe as an add-on to statin therapy. Additionally, LU codes are required to prescribe ezetimibe to older adults in our province, and this additional administrative requirement may have deterred practitioners from writing prescriptions for the therapy.

Further, cholesterol management in general has been reported to be suboptimal in high-risk patients [33–35], especially in older adults [33]. In a previous study of 396, 077 older adults (median age 75) with cardiovascular disease or diabetes in Ontario, only a small proportion were prescribed statins (*n* = 75, 617 patients, 19.1%) [33]. In our study, there were some patients who did not have lipid profiles checked nor see a family doctor or a specialist in a timely fashion after their discharge. This gap in follow-up care might have contributed to infrequent prescriptions.

Ezetimibe prescriptions may have also been low as between 2007 and 2015 there was controversy about the utility of this therapy in preventing clinically important cardiovascular outcomes. In 2008, the ENHANCE trial compared the effectiveness of simvastatin alone vs. simvastatin plus ezetimibe on the progression of atherosclerosis in patients with familial hypercholesterolemia, as measured by carotid intima-media thickness [12]. Although ezetimibe lowered LDL-C, it did not slow the progression of atherosclerosis over 24 months. In subsequent similar trials of ezetimibe, there was also no observed cardiovascular benefit of the therapy based upon surrogate imaging and arterial markers [13–15].

Evidence for a possible benefit of ezetimibe in reducing cardiovascular outcomes was not observed until 2011 in patients with renal impairment who participated in the SHARP trial [16]. In those who took simvastatin plus ezetimibe vs. placebo alone, cardiovascular risk was reduced by 17%. In the absence of a monotherapy arm however, it was impossible to definitively ascribe incremental benefit to ezetimibe. The IMPROVE-IT trial published in June 2015 also reported a benefit of ezetimibe in preventing major cardiovascular outcomes. In this study, when ezetimibe was prescribed with moderate-intensity simvastatin to patients with a recent myocardial infarction, there was a lower risk of cardiovascular outcomes in the ezetimibe group (32.7% with an event in ezetimibe plus simvastatin group vs. 34.7% in the simvastatin monotherapy group, absolute risk reduction 2%, *P* < 0.001) [10]. With the small effect size observed, missingness in the data, and controversy about the choice of the control arm (moderate-intensity statin therapy, specifically simvastatin), the clinical relevance of these results still remain controversial. In fact, The Endocrinology and Metabolic Drug Advisory committee of the US FDA has since voted against expanding the indication of ezetimibe for cardiovascular protection [36]. In our study, we did find that prescriptions for ezetimibe increased following its release on the provincial formulary in 2004, but appeared to decline after the publication of the ENHANCE trial. Ezetimibe prescriptions were also less common in the latter years of our study. A decline in the use of ezetimibe following ENHANCE has also been described in younger individuals with private drug coverage [37].

Interestingly, we also found that older men appeared less likely (23% decrease in odds) to be prescribed ezetimibe. Although there has been controversy about the impact of cholesterol on cardiovascular outcomes amongst the sexes [31], guidelines including the Second Adult Treatment Panel of the National Cholesterol Education Program and the Canadian Lipid Guidelines (both in force during our study’s time period) recommended similar cholesterol management in elderly women and men [27, 38, 39]. This difference was not apparent in the univariable analysis, but was significant in the multivariable analysis, implying a difference in the risk profiles between men and women.

Whatever the reasons for its infrequent use in high-risk patients in our region, our findings do suggest that efforts to examine the effectiveness of ezetimibe in preventing clinically important cardiovascular outcomes in Ontario would face a number of methodological challenges. With the limited number of prescriptions, proposed studies would likely suffer from low sample size and statistical power [40]. Further in routine care, patients did not stay on ezetimibe for very long (median 1.2 years, IQR 0.3 to 3.5 years), whereas in clinical trials, some took the therapy for up to 6 years. This would impact the attribution of ezetimibe to any observed outcomes. The choice of an appropriate control group would also be challenging due to potential confounding by indication (currently ezetimibe can only be prescribed with an appropriate LU code). Furthermore, changes in statin co-prescriptions and the initiation of other cardioprotective therapies during the follow-up period, would need to be considered and managed carefully in statistical analyses.

### Comparison with previous literature

To our best knowledge, the real-world use of ezetimibe in high-risk older adults with publically funded drug benefits has not been examined. In a younger group of Danish people with private drug benefits (2011–2012), those with a prior AMI, women, and those on higher-potency statins were more likely to be treated with ezetimibe [41]. Unlike in our study, patients with higher incomes were also more likely to be treated. This may be because in our country, lipid therapies are covered for older adults by our publically funded healthcare system.

### Strengths and weaknesses

Our study has several strengths. We examined patterns of ezetimibe use in a large group of representative, high-risk older adults in routine-care (*n* = 71, 125). We ascertained the demographics, medical comorbidities, medications and healthcare utilization of people who were prescribed the therapy, and detailed the predictors of a new prescription. We also noted some characteristics of new ezetimibe prescriptions.

There are some weaknesses to our study. Our results are not fully generalizable to younger populations with private drug benefits, nor to individuals who live outside of Ontario. The availability of data at the time of our study did not allow us to examine the impact of IMPROVE-IT on ezetimibe prescribing patterns. PCSK9 (proprotein convertase subtilisin-kexin type 9) inhibitors are also now available in our region, and can only be prescribed to people who have sub-target lipid control while taking both statins and ezetimibe. Since these medications did not enter the market until 2015, we could not evaluate their impact on ezetimibe prescribing. Further the data that we used were collected for administrative purposes, and were not specifically designed for this research study.

## Conclusions

Over the last decade in Ontario, only a small proportion of high-risk older adults received a prescription for ezetimibe within 6 months of an AMI. In this setting, a real-world drug effectiveness study would face a number of methodological challenges. Our work highlights the importance of examining drug prescription patterns prior to conducting drug effectiveness or safety studies in routine care.

## Additional files

Additional file 1: RECORD checklist of recommendations for the reporting of studies conducted using routinely collected health data. (DOCX 17 kb)

Additional file 2: Coding definitions for demographic characteristics and comorbidities. (DOCX 17 kb)

Additional file 3: Predictor variables for a new ezetimibe prescription. (DOCX 13 kb)

Additional file 4: Flow diagram of patient inclusion and exclusion into study cohort. (DOCX 25 kb)

Additional file 5: Percentage of older adults with a new ezetimibe prescription following a hospital encounter for an acute myocardial infarction. (DOCX 50 kb)

## Abbreviations

AMI: Acute myocardial infarction
CAPE: Client agency program enrolment database
CCI: Canadian classification of health interventions
CCP: Canadian classification of diagnostic, therapeutic and surgical procedures
DIN: Drug information number
FDA: Food and drug administration
HYPER: Hypertension database
ICD: International classification of diseases
ICES: Institute for Clinical Evaluative Sciences
IPDB: ICES physician database
IQR: Interquartile range
LDL-C: Low-density lipoprotein cholesterol
LU: Limited use
ODB: Ontario drug benefits
ODD: Ontario diabetes database
OHIP: Ontario Health Insurance Program
PCSK9: Proprotein convertase subtilisin-kexin type 9
SD: Standard deviation
