# Supplementary material for: Ezetimibe prescriptions in older Canadian adults after an acute myocardial infarction: a population-based cohort study
Source: Lipids Health Dis. 2018 Jan 8;17:8. doi: 10.1186/s12944-017-0649-5 (PMC5759247; doi:10.1186/s12944-017-0649-5)
Supplement: Supplementary file 1 — RECORD checklist of recommendations for the reporting of studies conducted using routinely collected health data. (DOCX 17 kb) [file 12944_2017_649_MOESM1_ESM.docx]

**Additional file 1. RECORD checklist of recommendations for the reporting of studies conducted using routinely collected health data**

|  | Item No | Recommendation | Reported |
| --- | --- | --- | --- |
| Title and abstract | 1 | 1.1 The type of data used should be specified in the title or abstract. When possible, the name of the databases should be included. | Abstract |
|  |  | 1.2 If applicable, the geographic region and time frame within which the study took place should be reported in the title or abstract. | Abstract |
|  |  | 1.3 If linkage between databases was conducted for the study, this should be clearly stated in the title or abstract | Abstract |
| Introduction | | |  |
| Background/rationale | 2 | Explain the scientific background and rationale for the investigation being reported | Introduction |
| Objectives | 3 | State specific objectives, including any pre-specified hypotheses | Introduction |
| Methods | | |  |
| Study design | 4 | Present key elements of study design early in the paper | Methods |
| Setting | 5 | Describe the setting, locations, and relevant dates, including periods of recruitment, exposure, follow-up, and data collection | Methods |
| Participants | 6 | 6.1 The methods of study population selection should be listed in detail. If this is not possible, an explanation should be provided. | Methods |
|  |  | 6.2 Any validation studies of the codes or algorithms used to select the population should be referenced. If validation was conducted for this study and not published elsewhere, detailed methods and results should be provided. | Methods |
|  |  | 6.3 If the study involved linkage of databases, consider use of a flow diagram or other graphical display to demonstrate the linkage process, including the number of individuals with linked data at each stage. | Additional file 4 |
| Variables | 7 | A complete list of codes and algorithms used to classify exposures, outcomes, confounders, and effect modifiers should be provided. If these cannot be reported, an explanation should be provided. | Methods, Additional file 2 |
| Data sources/ measurement | 8 | For each variable of interest, give sources of data and details of methods of assessment (measurement). Describe comparability of assessment methods if there is more than one group | Methods, Additional file 2 |
| Bias | 9 | Describe any efforts to address potential sources of bias | Methods |
| Study size | 10 | Explain how the study size was arrived at | Methods |
| Quantitative variables | 11 | Explain how quantitative variables were handled in the analyses. If applicable, describe which groupings were chosen and why | Table 1 |
| Statistical methods | 12 | 12.1 Describe all statistical methods, including those used to control for confounding | Methods |
|  |  | 12.2 Describe any methods used to examine subgroups and interactions | Not applicable |
|  |  | 12.3 Explain how missing data were addressed | Not applicable |
|  |  | 12.4 If applicable, explain how loss to follow-up was addressed | Not applicable |
|  |  | 12.5 Describe any sensitivity analyses | Not applicable |
| Data access and cleaning methods |  | 12.6 Authors should describe the extent to which the investigators had access to the database population used to create the study population. | Methods |
|  |  | 12.7 Authors should provide information on the data cleaning methods used in the study | Methods |
| Linkage |  | 12.8 State whether the study included person-level, institutional-level, or other data linkage across two or more databases. The methods of linkage and methods of linkage quality evaluation should be provided. | Methods |
| Results | | |  |
| Participants | 13 | 13.1 Describe in detail the selection of the persons included in the study (i.e. study population selection), including filtering based on data quality, data availability, and linkage. The selection of included persons can be described in the text and/or by means of the study flow diagram. | Results and Additional file 4 |
| Descriptive data | 14 | 14.1 Give characteristics of study participants (e.g. demographic, clinical, social) and information on exposures and potential confounders | Results, Table 1 |
|  |  | 14.2 Indicate number of participants with missing data for each variable of interest | Results |
|  |  | 14.3 Summarize follow-up time (e.g. average and total amount) | Results |
| Outcome data | 15 | Report numbers of outcome events or summary measures over time | Results, Tables 1 |
| Main results | 16 | 16.1 Give unadjusted estimates and, if applicable, confounder-adjusted estimates and their precision (e.g. 95% confidence interval). Make clear which confounders were adjusted for and why they were included | Results and Table 1 |
|  |  | 16.2 Report category boundaries when continuous variables were categorized | Table 1 |
|  |  | 16.3 If relevant, consider translating estimates of relative risk into absolute risk for a meaningful time period | Not applicable |
| Other analyses | 17 | Report other analyses done—e.g. analyses of subgroups and interactions, and sensitivity analyses | Not applicable |
| Discussion | | |  |
| Key results | 18 | Summarize key results with reference to study objectives | Discussion |
| Limitations | 19 | Discuss the implications of using data that were not created or collected to answer the specific research question(s). Include discussion of misclassification bias, unmeasured confounding, missing data and changing eligibility over time, as they pertain to the study being reported. | Discussion |
| Interpretation | 20 | Give a cautious overall interpretation of results considering objectives, limitations, multiplicity of analyses, results from similar studies, and other relevant evidence | Discussion |
| Generalizability | 21 | Discuss the generalizability (external validity) of the study results | Discussion |
| Other information | | |  |
| Funding | 22 | 22.1 Give the source of funding and the role of the funders for the present study and, if applicable, for the original study on which the present article is based | Acknowledgments |
| Accessibility of protocol, raw data and programming code |  | 22.2 Authors should provide information on how to access any supplemental information such as the study protocol, raw data, or programming code. | The data set from this study is held securely in coded form at the Institute for Clinical Evaluative Sciences (ICES). While data sharing agreements prohibit ICES from making the data set publicly available, access may be granted to those who meet pre-specified criteria for confidential access, available at www.ices.on.ca/DAS. |
