# Supplementary material for: Ezetimibe prescriptions in older Canadian adults after an acute myocardial infarction: a population-based cohort study
Source: Lipids Health Dis. 2018 Jan 8;17:8. doi: 10.1186/s12944-017-0649-5 (PMC5759247; doi:10.1186/s12944-017-0649-5)
Supplement: Supplementary file 2 — Coding definitions for demographic characteristics and comorbidities. (DOCX 17 kb) [file 12944_2017_649_MOESM2_ESM.docx]

**Additional file 2. Coding definitions for demographic characteristics and comorbidities**

| Characteristic | Database | Codes |
| --- | --- | --- |
| Age | RPDB |  |
| Sex | RPDB |  |
| Rural location | RPDB |  |
| Income quintile | Statistics Canada |  |
| Long term care status | ODB |  |
| Type of hospital | CIHI-DAD |  |
| Year of discharge | CIHI-DAD |  |
| Year of ezetimibe prescription | ODB |  |
| Family physician roster status | CAPE |  |
| Physician specialty | IPDB |  |
| Coronary artery disease (excluding angina) | CIHI-DAD  NACRS  OHIP | ICD 9: "412", "410", "411"  ICD 10: "I21", "I22", "Z955", "T822"  CCI: "1IJ50", "1IJ76"  CCP: "4801", "4802", "4803", "4804", "4805", "481", "482", "483"  OHIP FEE: "R741", "R742", "R743", "G298", "E646", "E651", "E652", "E654", "E655", "Z434", "Z448"  OHIP DX: "410", "412" |
| Stroke/Transient ischemic attack | CIHI-DAD  NACRS | ICD 9: "430", "431", "432", "434", "435", "436", "3623"  ICD 10: "I62", "I630", "I631", "I632", "I633", "I634", "I635", "I638", "I639", "I64", "H341", "I600", "I601", "I602", "I603", "I604", "I605", "I606", "I607", "I609", "I61", "G450", "G451", "G452", "G453", "G458", "G459", "H340" |
| Diabetes | ODD |  |
| Peripheral vascular disease | CIHI-DAD  OHIP | ICD 9: "4402", "4408", "4409", "5571", "4439", "444"  ICD 10: "I700", "I702", "I708", "I709", "I731", "I738", "I739", "K551"  CCP: "5125", "5129", "5014", "5016", "5018", "5028", "5038", "5126", "5159"  CCI: "1KA76", "1KA50", "1KE76", "1KG50", "1KG57", "1KG76MI", "1KG87", "1IA87LA", "1IB87LA", "1IC87LA", "1ID87", "1KA87LA", "1KE57"  OHIP FEE: "R787", "R780", "R797", "R804", "R809", "R875", "R815", "R936", "R783", "R784","R785", "E626", "R814", "R786", "R937", "R860", "R861", "R855", "R856", "R933", "R934", "R791", "E672", "R794", "R813", "R867", "E649" |
| Chronic kidney disease | CIHI-DAD  NACRS  OHIP | ICD 9: "4030", "4031", "4039", "4040", "4041", "4049", "585", "586", "5888", "5889", "2504"  ICD 10: "E102", "E112", "E132", "E142", "I12", "I13", "N08", "N18", "N19"  OHIP DX: "403", "585" |
| Chronic dialysis | OHIP  CIHI-DAD | CCP: "5195", "6698"  CCI: "1PZ21"  OHIP FEE: "R849", "G323", "G325", "G326", "G860", "G862", "G865" "G863", "G866", "G330", "G331", "G332", "G333", "G861", "G082", "G083", "G085", "G090", "G091", "G092", "G093", "G094", "G095", "G096", "G294", "G295", "G864", "H540", "H740" |
| Hypertension | HYPER database |  |
| Liver disease | CIHI-DAD  NACRS  OHIP | ICD 9: "4561", "4562", "070", "5722", "5723", "5724", "5728", "573", "7824", "V026", "2750", "2751", "7891", "7895", "571"  ICD 10: "B16", "B17", "B18", "B19", "I85", "R17", "R18", "R160", "R162", "B942", "Z225", "E831", "E830", "K70", "K713", "K714", "K715", "K717", "K721", "K729", "K73", "K74", "K753", "K754", "K758", "K759", "K76", "K77"  OHIP DX: "571", "573", "070"  OHIP FEE: "Z551", "Z554" |
| Congestive heart failure | CIHI-DAD  NACRS  OHIP | ICD 9: "425", "5184", "514", "428"  ICD 10: "I500", "I501", "I509", "I255", "J81"  CCP: "4961", "4962", "4963", "4964"  CCI: "1HP53", "1HP55", "1HZ53GRFR", "1HZ53LAFR", "1HZ53SYFR"  OHIP FEE: "R701", "R702", "Z429"  OHIP DX: "428" |
| Coronary revascularization | CIHI-DAD  OHIP | CCP: "481", "482", "483", "480"  CCI: "1IJ50", "1IJ26", "IIJ27", "1IJ57", "1IJ76", "1IJ57GQ", "1IJ54GQAZ"  OHIP FEE: "R741", "R742", "R743", "E651", "E652", "E654", "E646", "G298", "Z434", "G262" |
| Charlson comorbidity index | Charlson macro |  |
| Lipid test | OHIP | OHIP FEE: "L055" |

Abbreviations: CIHI-DAD, Canadian Institute for Health Information Discharge Abstract Database; DX, Diagnostic Code; ICD 9, International Classification of Diseases 9^th^ Revision; ICD 10, International Statistical Classification of Diseases and Related Health Problems, 10th Revision; OHIP, Ontario Health Insurance Plan Database; NACRS, National Ambulatory Care Reporting System Database
