# Supplementary material for: Ezetimibe prescriptions in older Canadian adults after an acute myocardial infarction: a population-based cohort study
Source: Lipids Health Dis. 2018 Jan 8;17:8. doi: 10.1186/s12944-017-0649-5 (PMC5759247; doi:10.1186/s12944-017-0649-5)
Supplement: Supplementary file 3 — Predictor variables for a new ezetimibe prescription. (DOCX 13 kb) [file 12944_2017_649_MOESM3_ESM.docx]

**Additional file 3. Predictor variables for a new ezetimibe prescription**

| Demographic variables | Age  Sex  Income  Place of residence (i.e. from long-term care or community)  Location of residence (i.e. rural or urban location)  Family doctor roster status |
| --- | --- |
| AMI hospitalization variables | Year of hospitalization  Location of hospital (i.e. academic or community hospital) |
| Patient comorbidities (in the 5 years prior to their hospitalization) | Coronary artery disease  Stroke  Diabetes  Peripheral vascular disease  Chronic kidney disease  Chronic dialysis  Hypertension  Chronic liver disease  Congestive heart failure  Charlson comorbidity index |
| Use of statin therapy (in the 120 days prior to hospitalization) | Evidence of statin use  Statin intensity |
| Healthcare follow-up after hospitalization | Lipid test following discharge  Family physician visit within 30 days of discharge  Specialist visit within 60 days of discharge (i.e. endocrinologist, cardiologist, internist) |
