# Supplementary material for: Ezetimibe prescriptions in older Canadian adults after an acute myocardial infarction: a population-based cohort study
Source: Lipids Health Dis. 2018 Jan 8;17:8. doi: 10.1186/s12944-017-0649-5 (PMC5759247; doi:10.1186/s12944-017-0649-5)
Supplement: Supplementary file 4 — Flow diagram of patient inclusion and exclusion into study cohort. (DOCX 25 kb) [file 12944_2017_649_MOESM4_ESM.docx]

**Additional file 4. Flow diagram of patient inclusion and exclusion into study cohort**

Excluded:

Missing age, sex, age <66 n=71 462

Non-Ontario resident n=59

Death prior to discharge n=11 886

Evidence of prior AMI n=10 235
Prior ezetimibe prescription n=3050

Patients with a valid health card number and evidence of hospitalization for acute myocardial infarction from April 1, 2005 until March 31, 2014

n=167 817

No ezetimibe prescription within 6 months

of discharge

n=69 895

Total acute myocardial infarction cohort

n=71 125

Evidence of new ezetimibe prescription within 6 months of discharge

n=1230
