# Supplementary material for: Ezetimibe prescriptions in older Canadian adults after an acute myocardial infarction: a population-based cohort study
Source: Lipids Health Dis. 2018 Jan 8;17:8. doi: 10.1186/s12944-017-0649-5 (PMC5759247; doi:10.1186/s12944-017-0649-5)
Supplement: Supplementary file 5 — Percentage of older adults with a new ezetimibe prescription following a hospital encounter for an acute myocardial infarction. (DOCX 50 kb) [file 12944_2017_649_MOESM5_ESM.docx]

**Additional file 5. Percentage of older adults with a new ezetimibe prescription following a hospital encounter for an acute myocardial infarction**
